# ESOT Consensus Platform for Organ Transplantation: Setting the Stage for a Rigorous, Regularly Updated Development Process

**DOI:** 10.3389/ti.2022.10915

**Published:** 2022-10-24

**Authors:** Umberto Cillo, Annemarie Weissenbacher, Liset Pengel, Ina Jochmans, Daniele Roppolo, Cristiano Amarelli, Luca S. Belli, Marina Berenguer, Aiko De Vries, Joana Ferrer, John Friedewald, Lucrezia Furian, Sharlene Greenwood, Diethard Monbaliu, Silvio Nadalin, Arne Neyrinck, Mario Strazzabosco, Christian Toso, Gianluigi Zaza, Raj Thuraisingham, Thierry Berney, Luciano Potena, Nuria Montserrat, Nazia Selzner

**Affiliations:** ^1^ Chirurgia Generale 2, Epato-Bilio-Pancreatica e Centro Trapianto di Fegato, Azienda Ospedale Università Padova, Padova, Italy; ^2^ Department of Visceral, Transplant and Thoracic Surgery, Center of Operative Medicine, Medical University of Innsbruck, Innsbruck, Austria; ^3^ Centre for Evidence in Transplantation, Nuffield Department of Surgical Sciences, University of Oxford, Oxford, United Kingdom; ^4^ Transplantation Research Group, Department of Microbiology, Immunology, and Transplantation, KU Leuven, Leuven, Belgium; ^5^ Department of Abdominal Transplant Surgery, University Hospitals Leuven, Leuven, Belgium; ^6^ ESOT Office, Padova, Italy; ^7^ Ospedale Monaldi, Azienda dei Colli, Naples, Italy; ^8^ Hepatology and Gastroenterology Unit, ASST GOM Niguarda, Milan, Italy; ^9^ Liver Unit, Hospital Universitario y Politécnico La Fe, Valencia, Spain; ^10^ Division of Nephrology, Department of Medicine, Leiden Transplant Center, Leiden University Medical Center, Leiden University, Leiden, Netherlands; ^11^ Hepatobiliopancreatic Surgery and Liver and Pancreatic Transplantation Unit, Department of Surgery, Institute Clínic of Digestive and Metabolic Diseases (ICMDiM), Hospital Clínic, University of Barcelona, Barcelona, Spain; ^12^ Barcelona Clínic Liver Cancer Group (BCLC), University of Barcelona, Barcelona, Spain; ^13^ August Pi i Sunyer Biomedical, Research Institute (IDIBAPS), University of Barcelona, Barcelona, Spain; ^14^ Network for Biomedical Research in Hepatic and Digestive Diseases (CIBERehd), Barcelona, Spain; ^15^ Division of Medicine and Surgery, Northwestern University, Chicago, IL, United States; ^16^ Kidney and Pancreas Transplantation Unit, Department of Surgical, Oncological and Gastroenterological Sciences, University of Padua, Padua, Italy; ^17^ King’s College Hospital NHS Trust, London, UK King’s College London, London, United Kingdom; ^18^ Department of Abdominal Transplant Surgery, University Hospitals Leuven, Catholic University Leuven, Leuven, Belgium; ^19^ Department of General, Visceral and Transplant Surgery, University Hospital Tübingen, Tübingen, Germany; ^20^ Department of Anesthesiology, University Hospitals Leuven, Leuven, Belgium; ^21^ Department of Cardiovascular Sciences, Catholic University Leuven, Leuven, Belgium; ^22^ Liver Center, Department of Internal Medicine, School of Medicine, Yale University, New Haven, CT, United States; ^23^ Division of Abdominal Surgery, Geneva University Hospitals, Geneva, Switzerland; ^24^ Nephrology, Dialysis and Transplantation Unit, Department of Medical and Surgical Sciences, University of Foggia, Foggia, Italy; ^25^ Royal London Hospital, London, United Kingdom; ^26^ School of Medicine, University of Geneva, Geneva, Switzerland; ^27^ Ilia State University, Tbilisi, Georgia; ^28^ Heart Failure and Transplant Unit, IRCCS Azienda Ospedaliero-Universitaria di Bologna, Bologna, Italy; ^29^ Pluripotency for Organ Regeneration, Institute for Bioengineering of Catalonia (IBEC), The Barcelona Institute of Science and Technology (BIST), Barcelona, Spain; ^30^ Centro de Investigación Biomédica en Red en Bioingeniería, Biomateriales y Nanomedicina, Barcelona, Spain; ^31^ Institució Catalana de Recerca i Estudis Avançats (ICREA), Barcelona, Spain; ^32^ Ajmera Transplant Center, University of Toronto, Toronto, ON, Canada

**Keywords:** organ transplantation, methodology, guidelines, consensus conference, platform

## Abstract

The European Society for Organ Transplantation (ESOT) has created a platform for the development of rigorous and regularly updated evidence based guidelines for clinical practice in the transplantation field. A dedicated Guideline Taskforce, including ESOT-council members, a representative from the Centre for Evidence in Transplantation, editors of the journal Transplant International has developed transparent procedures to guide the development of guidelines, recommendations, and consensus statements. During ESOT’s first Consensus Conference in November 2022, leading experts will present in-depth evidence based reviews of nine themes and will propose recommendations aimed at reaching a consensus after public discussion and assessment by an independent jury. All recommendations and consensus statements produced for the nine selected topics will be published including the entire evidence-based consensus-finding process. An extensive literature review of each topic was conducted to provide final evidence and/or expert opinion.

## Introduction

High-quality, evidence-based clinical practice guidance documents to support best practice in solid organ transplantation along with improving the quality of life are increasingly needed. These are statements that include recommendations intended to optimize patient care, lead to better clinical outcomes, and improve cost effectiveness. Furthermore, they provide the opportunity to identify areas requiring further research and serve an educational scope. Clinical Practice Guideline statements are informed by a systematic review of evidence and an assessment of the benefits of alternative care options. The multidisciplinary and multiprocedural nature of organ transplantation, the intrinsic difficulty in designing and carrying out numerically and methodologically sound comparative studies, and the ever-changing landscape of knowledge and therapeutics, challenge the realization of a solid evidence framework in some crucial areas of the field. Solid organ transplants, therefore, more than other clinical areas, need implementation of a systematic, continuous expert work dedicated to guideline and consensus production to help clinicians with framing evidence and expert opinions into clinical practical approaches (1–3).

The European Society of Organ Transplantation (ESOT) is recently giving high priority to the development of clinical practice guidelines launching a structured and continuous dedicated action plan. In January 2022, ESOT created a guideline taskforce (GT) composed of ESOT leadership and Transplant International editorial board members. The GT has the fundamental commitment to promote methodologically homogeneous guideline and consensus activities and to warrant trustworthiness, transparency and continuity of the processes. Furthermore, the GT selects cutting edge topics, initiates and realizes consensus processes among experts, draws guidelines and promotes dissemination of the compiled products.

Guideline and consensus related material will undergo widespread dissemination within the transplant community through publications in Transplant International, ESOT congresses, and platforms as well as through networking *via* social media. Patients and their representatives will play an active role in the consensus development processes and will be targets of the dissemination activities according to the principles and concepts of value-based health care (VBHC). When appropriate, the GT will involve stakeholders including those in health care management and economics, organ sharing organizations, and health care policy makers.

Besides drafting a uniform methodology for ESOT guidance/guideline production and promoting topic selection, the GT created a platform for the development of methodologically solid and up-to-date evidence-based guidelines for clinical practice in the transplantation field. This platform guarantees procedural and logistical continuity to ESOT activities in the field of consensus processes and guideline production.

The first edition of the Transplant Learning Journey (TLJ) 3.0, after several months of preparatory work, is there to produce systematic reviews of evidence and to grade evidence followed by drafting and sharing recommendations. During TLJ 3.0 in Prague 13th–15th November 2022, the 3-day consensus conference, a series of consensus-based clinical guidance documents comprising research topics considered as cutting-edge will be established.

## Aims

The main purpose of the TLJ 3.0 ESOT GT and the consensus conference is to provide methodologically solid evidence-based and best-practice recommendations reflecting the latest knowledge.

While creating clinical guidance through expertise and knowledge from all stakeholders involved in organ transplantation within the ESOT community and beyond, a further goal is to provide resources in the form of reference databases on an available platform maintained and updated continuously to lead the way in organ transplantation.

The present report is intentionally submitted for publication and it will be freely available prior to TLJ 3.0 event, to make publicly available and report fully with trustworthiness and transparency (1, 2) the new course of ESOT guideline and consensus processes in organ transplantation. The aim is to disclose the methodology of the ESOT consensus platform from its conception to its development, in line with the principles of openness and transparency (1, 2), which are fundamental where relevant potential policy changes are expected. In that light, this report was submitted to Transplant International prior to the event.

## Methods

A dedicated ESOT GT established a methodologic action plan in January 2022 and elaborated a handbook formalizing the processes associated with the preparation of ESOT Clinical Practice Guidelines, including selection of topics for new guidelines, writing, reviewing, approval, dissemination, and update. The document also defines the governance of the process and the roles of the various committees. This handbook has been open to be consulted on the ESOT website since the end of September 2022.

In line with the established action plan, the ESOT GT launched the event “Transplant Learning Journey (TLJ) 3.0” as an in-person consensus conference, designed as a modified NIH (National Institute of Health) model consensus development conference (1–6). Such a consensus development process was organized in collaboration with ESOT sections ELITA, EKITA, EPITA, ECTTA, ETAHP, the Education Committee, and YPT. The ILTS collaborated as well for some specific topics.

The platform, and its future developments, will represent ESOT’s permanent operative tool to regularly elaborate and deliver rigorous and homogenous consensus statements and publications. Due to the known limitations related to face-to-face consensus conferences, particular attention has been given to methods for topic selection, selection and number of steering committee members, and review of evidence.

The Delphi method will be applied to arrive at a group opinion by surveying the expert panels including SC, conference attendees and jury members. The final result will reflect a solid consensus of experts in the field (7, 8).

In the setting of the ESOT TLJ consensus conferences, the Delphi method is an appropriate technique as it can help to come to a conclusion under several circumstances which have been described in the late 1970s already (9). When a topic, or facing a challenge, in transplantation is not perfectly suitable for precise objective analytical techniques but benefit from subjective experts’ opinions, Delphi rounds can be particularly useful to find consensus. This technique is also helpful and supportive to draw a conclusion when discussion participants cannot be brought together to have direct, face-to-face interactions and discussions for a variety of reasons (timing, costs, pandemic, etc.) and remote ± anonymous voting is needed (9). In the particular setting of TLJ 3.0, a public appraisal of the results the Delphi conducted study “ENGAGE” (European Giudelines for the Management of Graft Recipient Consensus Project) will be realized.

The Delphi method will also be applied to rediscuss and modify crucial recommendations if consensus will not be reached at TLJ 3.0.

### Topic Selection for the 2022 European Society for Organ Transplantation Consensus Conference

An open call for topic proposals was issued to ESOT Sections and Committees in January 2022. Overall, 25 topic proposals were received and sent out to all members of the GT who rated them individually at a first step according to following criteria: 1) rating the proposal from 1 to 10; 2) recommending the topic yes/no; 3) marking the proposed group members 1) good proposition, 2) good but unbalanced, i3) needs to be discussed.

In a joint meeting, the GT reviewed and prioritized all submitted proposals and selected nine that met the following criteria: 1) cutting edge topics for which a consensus would have an impact on healthcare; 2) lack of similar guidelines or recommendations for this topic or an urgent need for an update of a previous version; 3) identification of barriers or data gaps requiring consensus recommendations to progress the field; 4) feasibility in the context of TLJ 3.0 meeting including minimal availability of published evidence; 5) completion of previous activated ESOT consensus processes; 6) collaborative forum of European and international leaders to exchange experience and knowledge.

**FIGURE 1:**
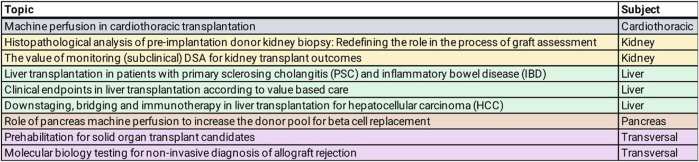
Topics selected by ESOT Guideline Taskforce (GT) for consensus conference, TLJ 3.0, Prague November 2022.

Figure 1 shows the nine topics selected by the GT and validated by the ESOT Executive Committee for the ESOT consensus conference during the TLJ 3.0 in Prague on November 13th–15th (10).

### Steering Committee Member Selection

For each of the selected topics, a specific steering committee (SC) was composed. The SC consists of a chair and co-chair, expert-members in the topic field, the Centre for Evidence in Transplantation (CET) (11), a YPT-representative working with the SC to collect and analyze the available topic-relevant literature, and a GT member to liaise with ESOT.

The GT had the final responsibility to nominate the SC members for each topic, though it did invite the topic proposers to suggest expert members. Depending on the balance of the proposed group representatives (expertise, gender, nationality etc., see below), the GT did either accept or request a modification of the member composition.

Each SC is led by a chair and a co-chair to warrant independency between topic proposers and guideline developers and to avoid bias and imbalances (12); selection of chair and co-chair followed a collaborative decision making process (GT and topic proposers) after exclusion of conflict of interests. The SC comprises of 8–14 members with a range of backgrounds to warrant a multidisciplinary expert discussion. In one case (Biomarker prediction in solid organ transplantation) the wide range of subtopics required a larger SC of 23 experts. When selecting SC members, consideration was given to: 1) representation of different disciplines and expertise; 2) gender balance; 3) broad geographic representation; 4) involvement of all health care professionals, if indicated and possible; 5) involvement of patient and public representatives if indicated; 6) involvement of members of ESOT YPT (young professionals in transplantation); 7) involvement of methodologists when indicated.

Some of the consensus topics are developed jointly with other international organizations. In those cases, representatives suggested by the partner organization were included as members of the SC and involved throughout the entire process.

The composition of the nine SC, including roles, is illustrated in Table 1.

**TABLE 1:** Composition of the nine steering committees (SC).

Topic: Machine perfusion in cardiothoracic transplantation
Chairs: Arne Neyrinck, Cristiano Amarelli
Steering committee: Clemens Aigner, Irene Bello, Massimo Boffini, Stephan Clark, Marita Dalvindt, Julien de Wolf, Stephan Ensminger, David Gomez de Antonio, Martin Schweiger, Sandro Sponga, Bettina Wiegmann
Topic: Histopathological analysis of pre-implantation donor kidney biopsy: Redefining the role in the process of graft assessment (Part 1)
Chairs: Lucrezia Furian, Gianluigi Zaza
Steering committee: Jan Becker, David Cucchiari, Aiko de Vries, Albino Eccher, Sandrine Florquins, Jesper Kers, Lorna Marson, Marion Rabant, Michele Rossini
Topic: The value of monitoring (subclinical) donor specific antibodies (DSAs) for kidney transplant outcomes
Chair: Aiko de Vries
Steering committee: Dominique Bertrand, Klemens Budde, Emanuele Cozzi, Anthony Dorling, Marie Paule Emonds, Covadonga López del Moral, Soufian Meziyerh, Dennis van den Broek
Topic: Liver transplantation in patients with primary sclerosing cholangitis (PSC) and inflammatory bowel disease (IBD)
Chairs: Luca Belli, Silvio Nadalin
Steering committee: Annika Bergquist, Marco Carbone, Eleonora De Martin, Andrea Della Penna, Pal Dag Line, Chiara Mazzarelli, James Neuberger, Palak Trivedi
Topic: Clinical endpoints in liver transplantation according to value based care
Chairs: Umberto Cillo, Mario Strazzabosco
Steering committee: Marco Carbone, Agostino Colli, Costantino Fondevila, Anna Forsberg, Lorenzo Mantovani, Sandor Mihaly, Alessandra Nardi, James Neuberger, Wojtek Polak, Karen Rockell, Ian Rowe, Liz Schick
Topic: Downstaging, bridging and immunotherapy in liver transplantation for HCC
Chair: Christian Toso
Steering committee: René Adam, Sherrie Bhoori, Umberto Cillo, Marco Claasen, Constantino Fondevilla, Bastiaan Rakke, Maria Reig, Gonzalo Sapisochin, Dimitri Sneiders, Parissa Tabrizian
Topic: Role of pancreas machine perfusion to increase the donor pool for beta cell replacement
Chair: Joana Ferrer
Steering committee: Julien Branchereau, Jason Doppenberg, Cinthia Drachenberg, Marten A Engelse, Paul Johnson, Henri G. D. Leuvenink, Benoît Mesnard, Franka Messner, Ann Etohan Ogbemudia, Vassilios Papalois, Trevor Reichman, Fabio Vistoli, Steve White
Topic: Prehabilitation for solid organ transplant candidates
Chairs: Diethard Monbaliu, Sharlene Greenwood
Steering committee: Coby Annema, Ellen Castle, Stefan De Smet, Pisana Ferrari, Tania Januadis- Ferreira, Joost Klaasen, Evangelia Kouidi, Sunita Mathur, Yasna Overloop, Maria José Perez Saez
Topic: Molecular biology testing for non-invasive diagnosis of allograft rejection
Group: heart, Chair: Luciano Potena
Steering committee: Ingvild Birschmann, Maria Crespo Leiro, Kiran Khush, Annamaria Minervini, Andrianna Nikolova, Javier Segovia
Group: kidney, Chair: John Friedewald
Steering committee: Dany Anglicheau, Oriol Bestard, Sook Park, Joana Sellares, Claire Tinel
Group: liver, Chair: Marina Berenguer
Steering committee: Eleonora de Martin, Amelia Heissheimer, Josh Levitsky, Alina Lutu, Valeria Mas, Nabeel Wahid, Haseeb Zubair

Steering committee members participate on a voluntary basis and are not paid for their contribution. Travel and accommodation costs for meetings are reimbursed according to the relevant ESOT travel and meetings policy.

### Consensus Questions, Evidence Review and Formulation of Recommendations

A number of virtual meetings were held by the SC to define the scope and aims of their topics and to work on their particular consensus process. Further meetings are scheduled in the upcoming months. Key issues were identified and implemented in the process to be worked on. The agreed clinical questions were formulated according to the PICO methodology (PICO = Population, Intervention, Comparator and Outcome) (13). All PICO questions are listed in Supplementary Appendix S1. In some cases (i.e., VBHC endpoints in liver transplantation), the strict PICO format was methodologically not applicable (see below). PICO eliminations will be decided upon full agreement during the open discussion that will precede the conference or in the context of the meeting itself. All these changes will be accurately recorded and reported to assure full transparency of the process.

Following the definition of the PICOs, for each topic, literature searches were developed by expert staff from the CET who have expertise in conducting systematic reviews. The searches were conducted in the Transplant Library, Medline, and Embase with or without a date limit (dates differed for each of the groups) and the exact search date of each search was recorded (and will be reported in each consensus-dedicated publication). Bibliographic searches consisted of a combination of Medical Subject Headings and keywords. Search terms and strategies will be provided in the specific topic related publications. Searches, excluding grey literature (some SC included congress abstracts upon request) and following removal of duplicate references, resulted in unique references which were selected for title/abstract screening. If titles/abstracts appeared relevant to the PICO question, corresponding full texts were acquired and reviewed for possible inclusion and interactive reading, and to support the development of consensus statements. Due to the breadth of topics included, a full systematic review process for article review was not performed at this time. Rather, titles and abstracts were reviewed by CET members.

PRISMA flowcharts describing the number of studies identified by the literature search and number of studies selected for inclusion in the consensus statement will appear in the following topic-specific publications.

A short summary of the evidence addressing each key question by the included studies was prepared in an evidence table. The workgroup proposed a recommendation for each key question, based on the quality of evidence rated using the GRADE approach, with high quality rated as A, medium quality as B, and low quality as C; very low quality of evidence was not considered. In particular, in the evaluation of the quality of evidence according to GRADE the following features were considered: study design, risk of bias, inconsistency, indirectness, imprecision, number of patients, effect, importance (14). Strength of recommendation was rated as 1 (strong) or 2 (weak).

### Jury Selection

The ESOT GT decided to maximize community involvement and inclusion of different perspectives while maintaining a high level of quality by assigning a panel to assess the documents prior to finalization. To establish these panels, an open call to attract jury members was launched in July 2022 *via* the ESOT webpage (15). Jury applicants register for the conference and specify their wish to be part of the recommendation voting process and the specific topic of interest. Jury member applicants’ CVs are subsequently evaluated by the GT before acceptance to ensure they have the necessary experience allowing them to fairly assess the recommendations. Furthermore, due to the focus on patient-centered medicine, patients and patients’ representatives are eligible to apply as jury members. Trainees will have the opportunity to follow the work of all included TLJ 3.0 panels as observers according to their particular interests (15). When jury members are appointed by the GT, conflicts of interests must be disclosed.

Jury members will receive the selected evidence as well as a preliminary version of the recommendations before the conference. They will be asked to provide the SC with comments and suggestions for potential changes and refinements before the start of the in-person meeting in Prague. In this way, a constructive discussion can take place during the face-to-face meeting.

### Consensus Format

Working groups will include SC members and jury members. Working group processes will consist of the following: 1) SC leaders will introduce and present their topic to an extended panel composed of all working group members in addition to conference participants registered to participate in the in-person consensus discussion; 2) a single SC member, will provide an overview of the evidence for each key question and present the proposed recommendations; 3) feedback will be provided by working group members and conference participants with particular attention to the generation of clear and concise consensus statements taking into account the suggestions emerged by the discussion 4) the following day the consensus recommendations will undergo the jury vote. Consensus will be considered achieved will be considered as reached if an agreement rate of >80% is achieved; topic lectures and proposed consensus statements will be presented to the entire TLJ 3.0 audience in a dedicated session on the last day of the in-person meeting in Prague.

Consensus conference participants are selected and distributed amongst the working groups by the GT members. Complete information including the list of consensus conference working group domains, processes regarding consensus conference participant selection, development and refinement of consensus statements, and modified Delphi methodology including consensus polling will be also reported in Transplant International after the face-to-face meeting in Prague.

### Validation Committee and AGREE

A validation committee, including experts in validation procedures, will be formed after the jury members have been finalized. Consensus and recommendations will be reviewed by experts in validation according to the AGREE II guidelines: Appraisal of guidelines for research and evaluation II (16, 17). The complete validation and appraisal process will be published in due course after the in-person meeting in Prague.

## Summary and Next Steps

The 2022 ESOT Consensus Conference, as part of TLJ 3.0, will be the first consensus and guideline conference initiated by ESOT covering the entire field of organ transplantation including organ-specific as well as cross-cutting, inter- and multidisciplinary topics. This in-person event represents the impetus for the foundation of an ongoing consensus, recommendation, and guideline production process which launches also a permanent area, like a standing committee, within ESOT. All guidelines and recommendations produced and published by ESOT and its involved representatives will undergo a continuous review process to stay up to date. Pre-meeting responsibilities and activities included constitution of a taskforce, steering committees and their working group members, opening of the jury applications and their selection process. The guideline development process started with the identification of the topics of interest, formulation of PICO questions and the identification of the relevant evidence.

The consensus conference during the TLJ 3.0 consists out of discussion session on statements and generating recommendations including Delphi rounds in some cases, as well as a voting and a discussion session, on the last day during the in-person meeting (10). The TLJ 3.0 program, however, also includes educational sessions training on guideline and consensus statement production.

All recommendations and consensus statements produced for the nine selected topics will be published including the entire evidence-based consensus-finding process.

## Abbreviations

AGREE, Appraisal of Guidelines for REsearch & Evaluation; CET, Centre for Evidence in Transplantation; ECTTA, European Cardio Thoracic Transplant Association; EKITA, European Kidney Transplant Association; ELITA, European Liver and Intestine Transplant Association; EPITA, European Pancreas and Islet Transplant Association; ESOT, European Society for Organ Transplantation; ETAHP, European Transplant Allied Healthcare Professionals; GRADE, Grading of Recommendations, Assessment, Development and Evaluation; GT, Guideline Taskforce; ILTS, International Liver Transplantation Society; NIH, National Institute of Health; PICO, Population, Intervention, Comparator and Outcome; PRISMA, Preferred Reporting Items for Systematic Reviews and Meta-Analyses; SC, Steering committee; TJL, Transplant Learning Journey; VBHC, Value-based health care; YPT, Young Professionals in Transplantation.
