# Supplementary material for: ESOT Consensus Platform for Organ Transplantation: Setting the Stage for a Rigorous, Regularly Updated Development Process
Source: Transpl Int. 2022 Oct 24;35:10915. doi: 10.3389/ti.2022.10915 (PMC9667481; doi:10.3389/ti.2022.10915)
Supplement: Supplementary file 1 [file DataSheet1.pdf]

Appendix 1 Preliminary list of PICO questions

<u>Topic: Machine perfusion in cardiothoracic transplantation</u>

1. In heart transplantation, for which heart should machine perfusion be performed?
2. In heart transplantation which protocol/perfusate/perfusion strategy for ex-vivo/ex-situ heart perfusion leads to:
   - the highest conversion rates to transplantation?
   - PGD-rate distribution after transplantation
   - the lowest rate of postoperative graft failure?
   - the best 30-day survival/90-day survival and 1 year survival
3. In heart transplantation, which biomarker / parameter is capable to predict the graft survival, graft function, primary non function(O) during ex vivo Heart perfusion?
4. In heart transplantation, which recipients should benefit from a heart assessed by machine perfusion:
   - Machine perfusion in LVAD
   - Machine perfusion in Redo
   - Machine perfusion in pediatric patients
   - Machine perfusion in ECMO
   - Machine perfusion in standard patients
5. In lung transplantation which type of lungs should be assessed?
   - Recipient's outcomes transplanted from uncontrolled DCD donors with and without ex vivo:
     - Mortality
     - PGD III incidence
     - rate of postoperatively prolonged ECMO support?
     - CLAD free survival
     - HLOS in ICU
     - HLOS
   - Recipient's outcomes transplanted from controlled DCD donors with and without ex vivo
     - Mortality
     - PGD III incidence
     - rate of postoperatively prolonged ECMO support?
     - CLAD free survival
     - HLOS in ICU
     - HLOS
   - Number of grafts from controlled DCD discarded after in vivo assessment vs. ex vivo
   - Number of grafts from uncontrolled DCD discarded after in vivo assessment vs ex vivo
   - Recipient's outcomes in order of ischemia time with and without ex vivo transport or assessment
6. In lung transplantation which type of lungs should be improved with EVLP?
   - Recipient's outcomes transplanted from grafts with low PaFi ratio with and without EVLP

    - Mortality
    - PGD III incidence
    - rate of postoperatively prolonged ECMO support?
    - CLAD free survival
    - HLOS in ICU
    - HLOS
  - Recipient's outcomes transplanted from grafts with pulmonal embolism with and without EVLP
    - Mortality
    - PGD III incidence
    - rate of postoperatively prolonged ECMO support?
    - CLAD free survival
    - HLOS in ICU
    - HLOS
  - Recipient's outcomes transplanted from grafts with infection with and without EVLP
    - Mortality
    - PGD III incidence
    - rate of postoperatively prolonged ECMO support?
    - CLAD free survival
    - HLOS in ICU
    - HLOS

7. In lung transplantation which protocol/perfusate/ventilation strategy for ex-vivo/ex-situ lung perfusion leads to:
   - the highest conversion rates to transplantation?
   - the best pO2/FiO2 ratio at 24 / 48 / 72 hours posttransplant?
   - PGD-rate distribution within first 72 hours after transplantation
   - the lowest rate of postoperatively prolonged ECMO / artificial ventilation support?
   - the best 30-day survival/90-day survival/long term CLAD free survival/graft survival/overall survival
   - the longest reported perfusion periods
8. In lung transplantation, which parameters should be used to determine graft quality during ex vivo lung perfusion
   - With Toronto protocol
   - With Lund protocol
   - With OCS system
   - With XPS system
   - With Lung Assist system
   - With Vivoline/LS1/LS2 system
9. In lung transplantation which type of recipient-allograft match should be assessed?
   - Recipient's Risk factors for PGD 3/72h
   - Recipient's mortality risk factor
   - Waitlist mortality risk factor
10. Have paediatrics recipients' good results after EVLP in terms of:
    - Mortality
    - PGD incidence

- rate of postoperatively prolonged ECMO support?
- CLAD free survival
- Risk factors fort mortality after EVLP

11. Which lung recipients should we assessed leads to:
    - Mortality
    - PGD incidence
    - rate of postoperatively prolonged ECMO support?
    - CLAD free survival
    - Risk factors fort mortality after EVLP

Topic: Histopathological analysis of pre-implantation donor kidney biopsy: Redefining the Role in the Process of Graft Assessment (Part 1)

1. For the evaluation of chronic lesions in ECD kidneys, is the needle core biopsy comparable/inferior/superior to wedge biopsy or punch biopsies (with a skin puncher as in PMID 22492825) in terms of representativity of the entire renal parenchyma?
2. For the evaluation of chronic lesions in ECD kidneys, is the frozen section comparable/inferior/ superior to paraffin embedded section in terms of reliability of the reading from pathologists?
3. For score assessment of pre-implantation kidney biopsy in the evaluation of ECD is the experienced renal pathologist comparable/inferior/superior to on-call pathologist in terms of reproducibility and accuracy of the histological report?
4. In the quantification of the chronic damage in ECD kidneys, is glomerulosclerosis more reproducible in comparison with other parameters (interstitial fibrosis, tubular atrophy, wall/lumen ratio, arteriolar hyalinosis)?
5. In the quantification of the chronic damage in ECD kidneys is measurement of histological variables with digital pathology comparable/inferior/superior if compared with light microscopy?
6. In the quantification of the chronic damage in ECD kidneys is measurement of histological variables with the aid of special stainings (PAS/Trichromic/Silver) comparable/inferior/superior if compared with H&Eosin alone?
7. For ECD is the histological assessment comparable/inferior/superior to demographics and clinical variables (e.g. KDPI, GFR) to predict graft survival, graft function, primary non-function?
8. In the quantification of the chronic damage in ECD kidneys, is glomerulosclerosis percentage more representative than other parameters (interstitial fibrosis, tubular atrophy, arteriolar hyalinosis and cv score) to predict the graft survival, graft function, primary non-function?
9. In the quantification of the chronic damage in ECD kidneys, are histological composite scores (e.g.Karpinski-Remuzzi, CADI score,..) more representative than single parameters (glomerulosclerosis, interstitial fibrosis, tubular atrophy, arteriolar narrowing, arteriosclerosis) to predict the graft survival, graft function, primary non function?
10. In the quantification of the chronic damage in ECD kidneys, is a combined clinical-histopathological score more representative than histological score alone to predict the graft survival, graft function, primary non function?

Topic: The value of monitoring (subclinical) donor specific antibodies (DSAs) for kidney transplant outcomes

1. Is there merit in routine posttransplant monitoring of subclinical HLA-DSA's for transplant outcomes?
2. What percentage of subclinical DSA's persist? (assay remains positive in (multiple) subsequent measurements)
3. What is the a-priori chance of diagnosing (early) rejection to warrant an 'indication' biopsy if subclinical DSA's are detected on routine monitoring? (or to what extent does it identify other clinical issues)?
4. In case of a confirming (positive) biopsy, is a follow-up biopsy to be recommended, and at what time? (One year after first biopsy?)
5. Are subclinical DSA's associated with poorer outcomes?
6. Are persistent subclinical DSA's associated with worsening of biopsy scores or outcome?
7. Is there evidence that treating subclinical DSA's leads to beneficial effects?
8. Does subclinical DSA more frequently recognize certain HLA-loci (DQ)?
9. Have certain risk factors or determinants been identified (e.g. preformed vs de novo DSA, pretransplant non-DSA (vPRA), MFI pre- or posttransplant, C1q binding, HLA-class/locus-specificity, etc) which modify the impact of subclinical DSA's on outcomes?
10. Does the DSA detection technique (One Lambda vs Immunocor) and/or the positivity criteria of these techniques have effect on the outcome of those with subclinical DSA?
11. Is there a (cost-effective) strategy for routine DSA monitoring?

Topic: Liver transplantation in patients with Primary sclerosing cholangitis (PSC) and inflammatory bowel disease (IBD)

1. Is the prophylactic use of rotating antibiotic for recurrent cholangitis safe in view of LT? (emergence of MDR bacteria)
2. When should PSC patients on the waiting list be treated with biliary stents?
3. Can we identify parameters that support the decision-making process of liver re-transplantation for PSC recurrence?
4. Can we develop a strategy to monitor PSC recipient of LT for disease recurrence?
5. Is pre-emptive LT indicated for high-grade dysplasia in suspicious strictures?
6. Is the MELD allocation system suitable for patients with PSC?
7. Optimal immunosuppression for patients transplanted with PSC:
   - What endpoints should be used?
   - What combinations are used mainly?
   - Impact of steroids
   - Monotherapy or combination therapy
   - Impact of age, possibly pregnancy, IBD, type and extent of IBD
8. In liver transplantation for PSC: duct-to-duct anastomosis vs to Roux-en-Y hepaticojejunostomy
9. Is the use of ECD (including DCD) in PSC associated with higher rate of NAS compared to other LT indications?
10. What are the best screening and surveillance strategies for IBD associated with PSC? (Screening for the presence of IBD, monitoring activity of IBD, bowel cancer surveillance in IBD in pre-, peri- and post-LT).

11. What is the optimum therapeutic approach for maintaining remission in IBD associated with PSC; pre-, peri- and post-transplant? (Safety and efficacy profiles of biologics, calcineurin inhibitors, anti-proliferatives and corticosteroids)
12. Colectomy in PSC-associated colitis: indications, timing and type, and the impact on liver-related outcomes.
    - When the colectomy is indicated?
    - What is the optimal timing for colectomy with regards native liver-related, graft-related, and overall survival?
    - How does the timing of colectomy (pre-, peri- or post-transplant) affect the incidence and risk of developing recurrent PSC and/or other graft-related complications?
    - What is the optimal timing for colectomy (pre-, peri- or post-transplant) with regards minimising peri-operative (peri-transplant) complications?
    - Does the type of colonic resection (i.e. restorative vs. non-restorative colectomy; ileal pouch anal anastomosis vs. ileorectal anastomosis vs. ileostomy alone) affect the outcomes listed in 1-3 above?
    - How does the timing of colectomy (pre-, peri- or post-transplant) affect peri-operative outcomes with regards the colonic resection procedure itself?

Topic: Clinical Endpoints in liver transplantation according to value-based care

1. Which is the best single measure to evaluate liver transplantation process as a whole from the VBC perspective?
2. In Liver transplant recipients which is the best tool to adjust for quality of life the life gain of liver transplantation?
3. When gain in life years or reduction in years lost are not available/calculable which is the best measure to describe the transplant process from a VBM perspective?
4. What is the most appropriate time frame to assess liver transplant outcomes from a VBM perspective?
5. In a setting with optimal potential candidate referral and listing process, which is a the best measure to evaluate the quality of waiting list management in a VBHM perspective?
6. Which is the best metrics to describe the quality of early postoperative course?
7. Which are the unmet needs in defining the critical PROMs and PREMs to be included in liver transplant "core" evaluation and clinical trial design?

Topic: Downstaging, bridging and immunotherapy in liver transplantation for HCC

1. Does bridging decrease waitlist drop-out?
2. Does bridging improve post-transplant survival?
3. What minimal expected waiting time is associated with an increased risk of drop-out and justifies the use of bridging?
4. Does the type of response to bridging have an impact on survival?
5. What is the best bridging treatment in patients with single HCC <3 cm in size?
6. What is the best downstaging treatment in patients with single HCC >5 cm in size?
7. Should all patients outside transplant criteria (all comers) be considered for transplantation after successful downstaging?

8. Should partial response be accepted as a successful downstaging?
9. Should patients with complete response of HCC macrovascular invasion be considered for liver transplantation?
10. Are patients on immunotherapy prior to liver transplantation at higher risk of rejection?
11. What is the best way to assess response to immunotherapy?
12. What is the safety of the combined treatment with immunotherapy and loco-regional therapy (LRT)?

<u>Topic: Role of Pancreas Machine Perfusion to Increase the Donor Pool for beta cell replacement</u>

Chair: Joana Ferrer

Steering Committee: Julien Branchereau, Jason Doppenberg, Cinthia Drachenberg, Marten A Engelse, Paul Johnson, Henri G. D. Leuvenink, Benoît Mesnard, Franka Messner, Ann Etohan Ogbemudia, Vassilios Papalois, Trevor Reichman, Fabio Vistoli, Steve White

<u>Topic: Prehabilitation for solid organ transplant candidates</u>

1. In solid organ transplant candidates, what is the evidence for exercise training pre-transplant?
2. In solid organ transplant candidates, what type(s) of exercises are recommended in the pre-transplant phase?
3. In solid organ transplant candidates, what are the outcomes relevant to exercise and physical activity that should be measured pre-transplant?
4. In solid organ transplant candidates, what is the evidence for nutritional support pre-transplant?
5. In solid organ transplant candidates, what type(s) of nutritional support are recommended in the pretransplant phase?
6. In solid organ transplant candidates, what are the outcomes relevant to nutritional support that should be measured pre-transplant?
7. In solid organ transplant candidates, what is the evidence for psychosocial interventions pre-transplant?
8. In solid organ transplant candidates, what type(s) of psychological interventions are recommended in the pretransplant phase?
9. In solid organ transplant candidates, what are the outcomes relevant to psychological interventions that should be measured pre-transplant?
10. In solid organ transplant candidates, what is the evidence for the feasibility of prehabilitation?

Topic: Molecular Biology Testing for Non-Invasive Diagnosis of Allograft Rejection

Group: heart

1. In heart transplant patients with stable graft function, is ddcfDNA (or GEP) a reliable surveillance tool for subclinical acute rejection monitoring, compared to endomyocardial biopsy?
2. In heart transplant patients, is ddcfDNA (or GEP) reliable surveillance strategy to monitor for cardiac allograft vasculopathy as compared with standard diagnostic methods?
3. In heart transplant patients with stable graft function, is ddcfDNA (or GEP) a reliable marker to stratify prognosis (or monitor the efficacy of therapy) as compared to standard clinical classifiers
4. In heart transplant patients with stable graft function, are cardiac biomarkers (NT-proBNP, BNP, Troponin) reliable surveillance tool for subclinical acute rejection monitoring, compared to endomyocardial biopsy?

Group: kidney

1. In kidney transplant patients with stable graft function, is dd-cfDNA a reliable diagnostic tool for subclinical acute rejection monitoring when compared with standard of care (eGFR/creatinine monitoring or surveillance biopsy)?
2. In kidney transplant patients with stable graft function, is blood gene expression profiling (GEP) a reliable diagnostic tool for subclinical acute rejection monitoring when compared with standard of care (eGFR/creatinine monitoring or surveillance biopsy)?
3. In kidney transplant patients with stable graft function, is urinary chemokine monitoring a reliable diagnostic tool for subclinical acute rejection monitoring when compared with standard of care (eGFR/creatinine monitoring or surveillance biopsy)?
4. In kidney transplant patients with acute allograft dysfunction, is dd-cfDNA a reliable diagnostic tool for acute rejection monitoring when compared with standard of care (eGFR/creatinine monitoring or for cause biopsy)?
5. In kidney transplant patients with acute allograft dysfunction, is blood gene expression profiling (GEP) a reliable diagnostic tool for clinical acute rejection monitoring when compared with standard of care (eGFR/creatinine monitoring or for cause biopsy)?
6. In kidney transplant patients with acute allograft dysfunction, is urine chemokine measurement a reliable diagnostic tool for clinical acute rejection monitoring when compared with standard of care (eGFR/creatinine monitoring or for cause biopsy)?

Group: liver

1. Can biomarkers be used to predict chronic kidney disease (CKD) in liver transplant recipients?
2. Can biomarkers be used to predict HCC recurrence in liver transplant recipients?
3. Can biomarkers be used to predict recurrent liver disease in liver transplant recipients?
4. Can biomarkers be used to safely wean IS (minimization and eventually full withdrawal)?
